# Spectral analysis of transient amplifiers for death–birth updating constructed from regular graphs

**DOI:** 10.1007/s00285-021-01609-y

**Published:** 2021-05-16

**Authors:** Hendrik Richter

**Affiliations:** HTWK Leipzig University of Applied Sciences, Leipzig, Germany

**Keywords:** Evolutionary dynamics, Transient Amplifiers, Regular graphs, Spectral analysis, 92D15, 05C90, 05C50, 05C75

## Abstract

A central question of evolutionary dynamics on graphs is whether or not a mutation introduced in a population of residents survives and eventually even spreads to the whole population, or becomes extinct. The outcome naturally depends on the fitness of the mutant and the rules by which mutants and residents may propagate on the network, but arguably the most determining factor is the network structure. Some structured networks are transient amplifiers. They increase for a certain fitness range the fixation probability of beneficial mutations as compared to a well-mixed population. We study a perturbation method for identifying transient amplifiers for death–birth updating. The method involves calculating the coalescence times of random walks on graphs and finding the vertex with the largest remeeting time. If the graph is perturbed by removing an edge from this vertex, there is a certain likelihood that the resulting perturbed graph is a transient amplifier. We test all pairwise nonisomorphic regular graphs up to a certain order and thus cover the whole structural range expressible by these graphs. For cubic and quartic regular graphs we find a sufficiently large number of transient amplifiers. For these networks we carry out a spectral analysis and show that the graphs from which transient amplifiers can be constructed share certain structural properties. Identifying spectral and structural properties may promote finding and designing such networks.

## Introduction

An important measure for the success of an initially rare mutant among a resident population on an evolutionary graph is the fixation probability of the mutation. The evolutionary dynamics associated with the mutant’s spread most likely depends on its fitness, with a beneficial mutant possessing a higher fitness than the resident individuals, while a deleterious mutant has a lower fitness. Previous works have shown that an evolutionary graph describing a structured population can be categorized with respect to the complete graph representing a well-mixed population by comparing their fixation probabilities (Allen and Tarnita 2014; Adlam et al. 2015; Hindersin and Traulsen 2015; Jamieson-Lane and Hauert 2015; Pavlogiannis et al. 2017). Compared to a well-mixed population, some graphs produce a higher fixation probability for a beneficial mutant, thus amplifying the effect of selection. For some other graphs we find the opposite with an increased fixation probability for a deleterious mutation, and thus suppressing selection. There are even reducers of fixation which lower the fixation probability of all mutants, regardless of whether beneficial or deleterious (Allen et al. 2020). Finally, there are transient amplifiers characterized by an increased fixation probability for some range of the mutant’s fitness, and also graphs that have multiple transitions between amplification and suppression (Alcalde Cuesta et al. 2018).

The mechanisms of amplification and suppression of selection have significant implications on our understanding of evolutionary dynamics of real biological populations. By these mechanisms the spatial population structure becomes influential on the balance between fitness-dependent selection and random drift. The influence of the spatial population structure on evolutionary dynamics has been shown, for instance, for cancer initiation and progression (Hindersin et al. 2016b; Komarova et al. 2003; Komarova 2006; Nowak et al. 2003; Vermeulen et al. 2013), ageing of tissues (Cannataro et al. 2016, 2017), the spread of infections (Ottino-Löffler et al. 2017a, b) and the microbial evolution of antibiotic resistance (Krieger et al. 2020).

Fixation probabilities are determined by the structure of the evolutionary graph, but also by the rules by which mutants and residents propagate on the graph. Thus, whether an evolutionary graph is a transient amplifier, or an amplifier, or a suppressor, or even a reducer, does not only depend on the graph but also on the updating rule describing the dynamics of mutants and residents. Two updating rules frequently studied are birth–death (Bd) updating and death–birth (dB) updating. As transient amplifiers provide a mechanism for shifting the balance between natural selection and genetic drift, and thus have considerable significance in evolutionary dynamics, they have been studied intensively (Alcalde Cuesta et al. 2017; Allen et al. 2020; Hindersin and Traulsen 2015; Pavlogiannis et al. 2018; Tkadlec et al. 2020; Adlam et al. 2015; Pavlogiannis et al. 2017; Monk 2018; Jamieson-Lane and Hauert 2015). In these works there are two main approaches for identifying (transient) amplifiers. One approach is to check a large number of random graphs, for instance Erdös–Rényi or Barabási–Albert graphs (Alcalde Cuesta et al. 2018; Hindersin and Traulsen 2015; Möller et al. 2019; Tkadlec et al. 2019). Another approach is to design candidate graphs out of structural aspects and considerations (Adlam et al. 2015; Jamieson-Lane and Hauert 2015; Pavlogiannis et al. 2017, 2018). An example for the first approach is a comprehensive numerical study checking a larger number of random graphs with $N\le 14$ vertices, which found a multitude of amplifiers of selection for Bd, but none for dB updating (Hindersin and Traulsen 2015). From these results it was assumed that either there are no amplifiers for dB, or they are very rare, at least for graphs with small order. Examples for the second approach involve, for instance, constructing arbitrarily strong amplifiers for star and comet graphs, particularly by additionally designing weights (Pavlogiannis et al. 2017, 2018).

There are two recent works on amplifiers for dB updating upon which this work is particularly based. In Tkadlec et al. (2020) it is shown that for dB updating no universal amplification is possible and at most evolutionary graphs can be transient amplifiers. In Allen et al. (2020) a method is devised to check whether or not a given graph is at least an amplifier for weak selection. This method involves calculating the coalescence times of random walks on the graph (Allen et al. 2017) and finding the vertex with the largest remeeting time. If subsequently the graph is perturbed by removing an edge from this vertex, there is a certain likelihood that the resulting perturbed graph is a transient amplifier.

In principle, any graph can be tested by the perturbation method, but in this paper it is proposed to take as an input to the method all pairwise nonisomorphic *k*-regular graphs up to a certain order. A particular focus is on cubic and quartic regular graphs. This has two reasons. First, for a small order *N* the exact number of connected (pairwise nonisomorphic) regular graphs is known and all graphs can be generated algorithmically (Meringer 1999). With the available numerical resources the main results have been obtained for $N\le 22$ for cubic and $N\le 16$ for quartic graphs. Thus, the study is conducted for the whole set of these regular graphs and ensures that the whole structural range expressible by these graphs is covered. The second reason is that for graphs with higher degree than quintic graphs the method ceases to find transient amplifiers. By perturbing cubic and quartic regular graphs, however, we obtain a substantial number of graphs which construct transient amplifiers. Thus, the method discussed in the paper yields a sufficiently large set of amplifier graphs, which suggests to consider these graphs as an ensemble to be treated statistically. Moreover, we study structural graph properties by the spectrum of the normalized Laplacian, thus adding to the applications of spectral analysis of evolutionary graphs (Allen et al. 2019; Richter 2017, 2019a, b, 2020). In the analysis we use a smoothed spectral density which convolves the eigenvalues with a Gaussian kernel and show that those cubic or quartic regular graphs which allow to construct transient amplifiers have a characteristic spectral density curve. Thus, it becomes feasible to deduce from the spectrum of the graph if a transient amplifier can be constructed.

## Methods

### Constructing transient amplifiers

Our topic is evolutionary dynamics on graphs, and we place a population of *N* individuals on an undirected (and unweighted) graph ${\mathcal {G}}=(V,E)$ with vertices $v_i \in V$ and edges $e_{ij}\in E$. Each individual is represented by a vertex $v_i$ and an edge $e_{ij}=e_{ji}=1$ shows that the individuals associated with $v_i$ and $v_j$ are mutually interacting neighbors (Allen et al. 2017; Lieberman et al. 2005; Ohtsuki et al. 2007; Pattni et al. 2015; Richter 2017). We consider the graph ${\mathcal {G}}$ to be simple and connected with each vertex $v_i$ having degree $k_i$, which implies that there is no self-replacement and an individual on $v_i$ has $k_i$ neighbors. The test set used as an input to the perturbation method for constructing transient amplifiers puts a focus on cubic and quartic regular graphs, for which consequently there is $k_i=k=\{3,4\}$ for all vertices $v_i$.

Individuals can be of two types, mutants and residents. Residents have a constant fitness normalized to unity, while mutants have a fitness $r>0$. An individual can change from mutant to resident (and back) in a fitness-dependent selection process. We consider a death–birth (dB) process, e.g. Allen and Nowak (2014) and Pattni et al. (2015). The type of a vertex becomes vacant as the occupying individual is assumed to die, which happens uniformly at random. One of the neighbors is chosen to give birth with a probability depending on its fitness. It hands over its type, thus effectively replacing the death individual.

We are interested in the fixation probability $\varrho _{{\mathcal {G}}}$ defined as the expected probability that starting with a single mutant appearing at a vertex uniformly at random all vertices of the graph ${\mathcal {G}}$ eventually become the mutant type. In particular, we want to know how this fixation probability $\varrho _{{\mathcal {G}}}(r)$ compares to the fixation probability $\varrho _{{\mathcal {N}}}(r)$ for the complete graph with *N* vertices for varying fitness *r*. More specifically, a graph ${\mathcal {G}}$ is called an amplifier of selection if $\varrho _{{\mathcal {G}}}(r)<\varrho _{{\mathcal {N}}}(r)$ for $0<r<1$ and $\varrho _{{\mathcal {G}}}(r)>\varrho _{{\mathcal {N}}}(r)$ for $r>1$. Correspondingly, a graph ${\mathcal {G}}$ is a suppressor of selection if $\varrho _{{\mathcal {G}}}(r)>\varrho _{{\mathcal {N}}}(r)$ for $0<r<1$ and $\varrho _{{\mathcal {G}}}(r)<\varrho _{{\mathcal {N}}}(r)$ for $r>1$. Finally, a transient amplifier is characterized by $\varrho _{{\mathcal {G}}}(r)<\varrho _{{\mathcal {N}}}(r)$ for $r_{min}<r<1$ and $r>r_{max}$, while there also is $\varrho _{{\mathcal {G}}}(r)>\varrho _{{\mathcal {N}}}(r)$ for $1<r<r_{max}$ and some $0<r_{min}<1<r_{max}<\infty$ (Allen et al. 2020; Hindersin and Traulsen 2015; Adlam et al. 2015; Pavlogiannis et al. 2017).

There are two recent works on amplifiers for dB which inspired this work. In Tkadlec et al. (2020) it is shown that for dB updating no universal amplification is possible and at most evolutionary graphs can be transient amplifiers. In Allen et al. (2020) it is demonstrated that for weak selection, that is $r=1+\delta$ with $\delta \rightarrow 0$, the question of whether or not a graph ${\mathcal {G}}$ is an amplifier can be answered by a numerical test executable with polynomial time complexity. The test relies upon coalescing random walks (Allen et al. 2017) and involves calculating the effective population size $N_{eff}$ from the relative degree $\pi _i=k_i/\sum _{j \in {\mathcal {G}}} k_j$ and the remeeting time $\tau _i$ of vertex $v_i$. Thus, we have (Allen et al. 2020)

1 $$\begin{aligned} N_{eff}= \sum _{i \in {\mathcal {G}}} \pi _i \tau _i. \end{aligned}$$

The remeeting time $\tau _i$ can be obtained via

2 $$\begin{aligned} \tau _i= 1+ \sum _{j \in {\mathcal {G}}} p_{ij}\tau _{ij} \end{aligned}$$

from the coalescence times $\tau _{ij}$ and the step probabilities $p_{ij}=e_{ij}/k_i$ (implying $p_{ij}=1/k_i$, if $e_{ij}=1$ and $p_{ij}=0$ else). The remeeting times observe the condition $\sum _{i \in {\mathcal {G}}} \pi _{i} ^2\tau _{i}^{}=1$. Finally, the coalescence times $\tau _{ij}$ can be computed by solving the system of $$\left( {\begin{matrix}N\\ 2\end{matrix}}\right) $$ linear equations

3 $$\begin{aligned} \tau _{ij}= \left\{ \begin{array}{ll} 0 &{} \quad i=j\\ 1+ \frac{1}{2} \sum _{k \in {\mathcal {G}}} (p_{ik}\tau _{jk}+p_{jk}\tau _{ik}) &{} \quad i\ne j \end{array} \right. . \end{aligned}$$

In Allen et al. (2020) it is shown that a graph ${\mathcal {G}}$ is an amplifier of weak selection if

4 $$\begin{aligned} N_{eff}>N. \end{aligned}$$

Furthermore, it is argued that an amplifier of weak selection can be constructed by the following perturbation method. If the graph ${\mathcal {G}}$ is *k*-regular, then $k_i=k$ and $\pi _i=1/N$ for all $i=1,2,\ldots ,N$. Thus, with the identity condition $\sum _{i \in {\mathcal {G}}} \pi _{i} ^2\tau _{i}^{}=1$, we get from Eq. (1):

5 $$\begin{aligned} N_{eff}= \sum _{i \in {\mathcal {G}}} \tau _i/N=N \sum _{i \in {\mathcal {G}}} \pi _{i} ^2\tau _{i}^{}=N. \end{aligned}$$

According to Eq. (4), $N_{eff}=N$ confirms that *k*-regular graphs cannot be amplifiers of weak selection, but disturbing the regularity can change the equality. Moreover, most promising for such a perturbation is to remove a single (or even more than one) edge from the vertex $v_i$ with the largest remeeting time $\tau _i$, that is $\max (\tau _i)={\max }_{i \in {\mathcal {G}}} \, \tau _i$, on the *k*-regular graph to be tested. The argument is that if we take a regular graph and induce a small perturbation by removing an edge, the relative degree $\pi _i$ and the remeeting time $\tau _i$ experience small deviations $\varDelta \pi _i$ and $\varDelta \tau _i$. For the identity condition $\sum _{i \in {\mathcal {G}}} \pi _{i} ^2\tau _{i}^{}=1$ it follows

6 $$\begin{aligned} \sum _{i \in {\mathcal {G}}} \varDelta \big (\pi _{i} ^2\tau _{i}^{}\big )= \sum _{i \in {\mathcal {G}}} \big (2 \pi _i \varDelta \pi _{i} \tau _{i}^{}+ \pi _{i} ^2 \varDelta \tau _{i}^{}\big ) \approx 0. \end{aligned}$$

As there is $\pi _i=1/N$ for the unperturbed regular graph, we obtain

7 $$\begin{aligned} 1/N \sum _{i \in {\mathcal {G}}} \varDelta \tau _{i}^{} \approx -2 \sum _{i \in {\mathcal {G}}} \varDelta \pi _{i} \tau _{i}^{}. \end{aligned}$$

For the effective population size, Eq. (1), the perturbation yields

8 $$\begin{aligned} \varDelta N_{eff}= \sum _{i \in {\mathcal {G}}} \varDelta (\pi _i \tau _i) \approx \sum _{i \in {\mathcal {G}}} (\varDelta \pi _i \tau _i+\pi _i \varDelta \tau _i). \end{aligned}$$

By observing $\pi _i=1/N$ for the unperturbed regular graph and inserting Eq. (7), we get

9 $$\begin{aligned} \varDelta N_{eff} \approx -\sum _{i \in {\mathcal {G}}} \varDelta \pi _i \tau _i. \end{aligned}$$

This relationship implies that a positive perturbation of the effective population size (and thus the possibility to get $N_{eff}>N$) is obtained if for a large $\tau _i$ the perturbation induces a decrease of the relative degree $\pi _i$, that is a negative $\varDelta \pi _i$.

The perturbation method for finding transient amplifiers applies for weak selection, where $r=1+\delta$ and $\delta \rightarrow 0$. To find the range $1< r < r_{max}$ for which a graph is a transient amplifier for dB updating requires to calculate the fixation probabilities $\varrho _{{\mathcal {G}}}(r)$ and compare them to the fixation probability of the complete graph with *N* vertices. The fixation probabilities $\varrho _{{\mathcal {G}}}(r)$ analyzed in this paper are calculated by evaluating a Markov state transition matrix (Hindersin and Traulsen 2015; Hindersin et al. 2016a, 2019). This method is an exact computation and does not involve Monte Carlo simulations. Such Monte Carlo simulations rely upon repeating a numerical experiment (Broom et al. 2009; Hindersin et al. 2019). A mutant with given fitness *r* is placed randomly on a vertex of the graph. The evolutionary dynamics specified by the dB updating process leads for a sufficiently large number of iterations to either the mutant taking over the entire graph, or the mutant becoming extinct. The simulation is repeated many times, which yields a relative frequency of the mutation getting fixated, which in turn is interpreted as the fixation probability. Such a numerical procedure involves a large number of repetitions until an acceptable precision is obtained, and convergence to the fixation probability is sometimes difficult to ascertain (Hindersin et al. 2019). For the exact numerical calculation method used here there are no repetitions and no convergence issues. Apart from the problem of numerical round-off, the method yields an exact value. The limitation of the method is that it requires to solve a system of $2^N-2$ linear equations, which becomes infeasible for *N* getting large. For $N\le 22$, however, the calculations are still possible.

As a transient amplifier is characterized by $\varrho _{{\mathcal {G}}}(r)>\varrho _{{\mathcal {N}}}(r)$ for $1<r<r_{max}$, we need to compare the calculated $\varrho _{{\mathcal {G}}}(r)$ to the fixation probability of the complete graph $\varrho _{{\mathcal {N}}}(r)$, for which there is an analytic description (Kaveh et al. 2015; Hindersin and Traulsen 2015; Allen et al. 2020)

10 $$\begin{aligned} \varrho _{{\mathcal {N}}}(r)=\frac{N-1}{N} \frac{1-r^{-1}}{1-r^{-(N-1)}}. \end{aligned}$$

There is also an analytic description of the fixation probability for the cycle graph

11 $$\begin{aligned} \varrho _{{\mathcal {C}}}(r)= \frac{2(r-1)}{3r-1+r^{-(N-3)}-3r^{-(N-2)}} \end{aligned}$$

which we use to test the accuracy of the numerical calculation, see the Appendix, Fig. 11.

### Spectral analysis

For the spectral analysis of evolutionary graphs we consider the spectrum of the normalized Laplacian $L_{{\mathcal {G}}}$ of the graph ${\mathcal {G}}$, which is $L_{{\mathcal {G}}}=I-D^{-1/2}AD^{-1/2}$. The adjacency matrix of the graph ${\mathcal {G}}$ is *A* and *D* is the degree matrix. The spectrum is denoted by $\lambda ({\mathcal {G}})$ and consists of *N* eigenvalues $0=\lambda _1\le \lambda _2 \le \ldots \lambda _N\le 2$. The spectrum of the normalized Laplacian is used rather than the spectrum of the adjacency matrix as it has been shown that it captures well some geometric and structural properties (Banerjee and Jost 2008, 2009; Banerjee 2012; Gu et al. 2016). In addition, the spectrum is contained in the interval [0, 2] for any graph order and degree, which makes is convenient for comparing over varying order and degree. From the spectrum a spectral distance *d* can be defined, which can be used to compare two families (or classes) of graphs $({\mathcal {G}})$ and $({\mathcal {G}}')$. For each family only containing a single member, we can also compare two graphs ${\mathcal {G}}$ and ${\mathcal {G}}'$. The comparison can be done directly by viewing the histograms of the eigenvalue distribution $f_{({\mathcal {G}})}(x)$ on a discrete variable *X* as spectral plots (Banerjee and Jost 2007). A more refined comparison can be achieved by considering a smoothed spectral density which convolves the eigenvalues $\lambda _i$ with a Gaussian kernel with standard deviation $\sigma$ (Banerjee and Jost 2009; Banerjee 2012; Gu et al. 2016)

12 $$\begin{aligned} \varphi _{{\mathcal {G}}}(x)= \sum _{i=1}^{N} \frac{1}{\sqrt{2 \pi \sigma ^2}} \exp {\left( \frac{(x-\lambda _i)^2}{2 \sigma ^2} \right) }. \end{aligned}$$

We set $\sigma =1/(3N)$. From this continuous spectral density we can define a pseudometric on graphs by the distance (Gu et al. 2016)

13 $$\begin{aligned} d({\mathcal {G}},{\mathcal {G}}')= \int _0^2|\varphi _{{\mathcal {G}}}(x)-\varphi _{{\mathcal {G}}'}(x)|dx. \end{aligned}$$

An alternative way of characterizing Laplacian spectra is based on entropic graph measures. We consider the von Neumann entropy (Du et al. 2010; Feng et al. 2019), also known as Laplacian graph entropy, which recently found notable applications in complex networks analysis and pattern recognition (Passerini and Severini 2009; Han et al. 2012). We normalize $\nu _i=\lambda _i/N$ and obtain

14 $$\begin{aligned} S_{\mathcal {G}}=-\sum _{i=1}^N \nu _i \log _2 \nu _i \end{aligned}$$

with $0 \log _2 0 =0$, by convention.

## Results

### Regular graphs as amplifier constructors

Previous approaches to finding amplifiers of selection for dB updating focused on checking numerically generated random graphs, for instance Erdös–Rényi or Barabási–Albert graphs with prescribed expected degree or linking number (Alcalde Cuesta et al. 2018; Hindersin and Traulsen 2015; Möller et al. 2019; Tkadlec et al. 2019). As we are interested in how population structure relates to evolutionary dynamics, it would be most desirable to study differences in the graph structure among the realizations of random graphs. Ideally, these structural differences would encompass all what is structurally possible. There are, however, some problems with such an approach. Suppose we generate random graphs and two of them are structurally the same. But in an experimental setting this might be hard to detect as the computational problem of determining whether two finite graphs are isomorphic is not solvable in polynomial time (Arvind and Torán 2005; Babai 2019). On the other hand, isomorphic graphs have the same fixation properties. Related to the problem addresses in this paper, the numerical procedure would yield for two random but isomorphic graphs the same $N_{eff}$. Moreover, even for a relatively small number of vertices, the number of nominally different random graphs is huge, see for instance the results for the class of labelled regular graphs (Wormald 1999). In addition, algorithms producing random graphs, regular or otherwise, may have a bias towards certain graph structures (Bayati et al. 2010; Klein-Hennig and Hartmann 2012). Consequently, even if a large number of random graphs is produced and checked, there might be isomorphic graphs that have the same structural properties or there might be “blind spots” for certain structural types of graphs. Thus, as long as not all graphs of a certain class are enumerated, it is far from certain that by checking a finite number of random graphs from this class the relevant search space of graph structures has been adequately covered.

The numerical procedure suggested here aims for a more systematically conducted search for a certain class of graphs. For a small number of vertices *N* the exact number of connected (pairwise nonisomorphic) *k*-regular graphs is known. This particularly applies for cubic and quartic graphs, see e.g. Meringer (1999) and WolframMathWorld (2021) and also Table 2 in the Appendix. With the numerical resources available for this study cubic graphs could be checked for $N \le 22$ and quartic graphs for $N \le 16$. The complete set is tested. Thus, the study ensures that the whole structural range of these regular graphs is covered. We take these graphs as the input for the perturbation method described in the previous section to find transient amplifiers of weak selection.

**Table 1:** The number ${\mathcal {A}}_k(N)$ of simple, connected, pairwise nonisomorphic *k*-regular graphs on *N* vertices that construct transient amplifiers, also called amplifier constructors

*N*	k			
	3	4	5	6-12
11		1		0
12	1	4	0	0
13		23		0
14	7	108 / (3)	30 / (5)	0
15		562 / (16)		
16	42	3.129 / (36)		
18	265			
20	1.822			
22	13.889			

Compare to the total number of nonisomorphic *k*-regular graphs on *N* vertices in Table 2 of the Appendix. The numbers in parenthesis are the graphs for which 2 edges can be removed to produce an amplifier

**Fig. 1:**
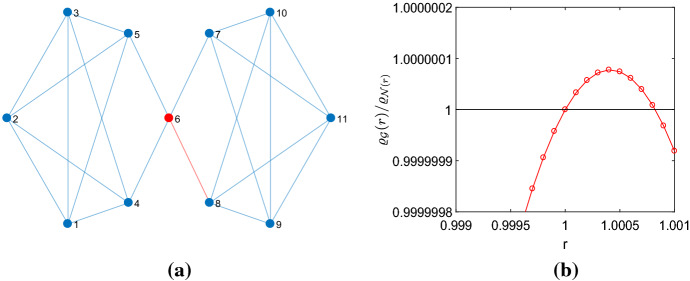
The 4-regular graph of order 11 used to construct a transient amplifier for $1<r<r_{max}\approx 1.00075$. **a** The vertex $v_6$ has the maximal remeeting time $\tau _6=17.6997$ and a transient amplifier arises if an edge from this vertex is removed. Due to the symmetry removing any of the 4 edges yields a graph with $N_{eff}=11.0008>11$. **b** The ratio between the fixation probability of the graph ${\mathcal {G}}$ and the complete graph which indicates a transient amplifier for $\varrho _{{\mathcal {G}}}(r)/\varrho _{{\mathcal {N}}}(r)>1$

Table 1 gives the numbers ${\mathcal {A}}_k(N)$ of regular graphs that produce $N_{eff}>N$ if the perturbation method is applied. We call these graphs amplifier constructors. Apart from the cubic and quartic graphs, also the graphs with degree $4<k<N-2$ have been tested for $N\le 14$. Viewing these results, several observations can be made. Within the bounds of the experimental setting, only for $k=\{3,4,5\}$ regular graphs produce transient amplifiers. For the tested graphs with $k\ge 6$ no such graphs have been found for $N\le 14$. It is, however, quite possible that $N\le 14$ is not large enough because a certain difference $(N-k)$ is needed for a regular graph having the property to be an amplifier constructor. Another possibility is that for graphs with a higher degree *k* the perturbation method only gives transient amplifiers if a larger number of edges is removed. This should to be clarified by future work. The smallest graph for which $N_{eff}>N$ was found is the quartic graph with order $N=11$ given in Fig. 1a. Although the results given in this paper apply to unlabeled pairwise nonisomorphic graphs, the vertices of the graph in Fig. 1a, as well as the vertices of graphs in other figures, are labeled with consecutive integers. This is solely done to ease addressing and communicating certain vertices or edges, for instance for indicating which vertex has the largest remeeting time, or which edge is removed from a given graph.

A second observation is that the absolute number ${\mathcal {A}}_4(N)$ is larger than ${\mathcal {A}}_3(N)$. But as ${\mathcal {L}}_4(N)$ grows much faster than ${\mathcal {L}}_3(N)$, compare Table 2 in the Appendix, the relative number is not. For cubic graphs, we have a ratio ${\mathcal {A}}_3(N)/{\mathcal {L}}_3(N)= \{ \frac{1}{85},\frac{7}{509},\frac{42}{4.060}, \frac{265}{41.301}, \frac{1.822}{510.489}, \frac{13.889}{7.319.447}\}$ for $N=\{12,14,\ldots ,22\}$, which shows that the percentage of regular graphs with amplifier construction properties starts from more than $1\%$ for $N=12$ to go to $0.35\%$ and $0.19\%$ for $N=\{20,22\}$. For quartic graphs with $N=\{11,12,\ldots ,16 \}$ the ratio ${\mathcal {A}}_4(N)/{\mathcal {L}}_4(N)$ sets out with $0.37\%$ for $N=11$ to end with $0.07\%$ and $0.04\%$ for $N=\{15,16\}$. In other words, regular graphs that construct transient amplifiers are rare but not unprecedented.

Table 1 gives the numbers of graphs from which at least one transient amplifier graph can be constructed. As cubic graphs can be perturbed by removing 3 edges from the vertex with the largest remeeting time, frequently from the same graph more than one amplifier is obtained. Sometimes, the resulting amplifiers are the same due to symmetry (as for instance the graph in Fig. 1a), but there are also cases where transient amplifiers with different $N_{eff}$ arise. The same applies for quartic graphs, which additionally have the property that a small number of these graphs allows to remove 2 edges to produce an amplifier. This has been observed for $14 \le N \le 16$ and also for the quintic graphs with $N=14$. Table  1 gives the number of these graphs with 2 removable edges as the quantities in parenthesis.

**Fig. 2:**
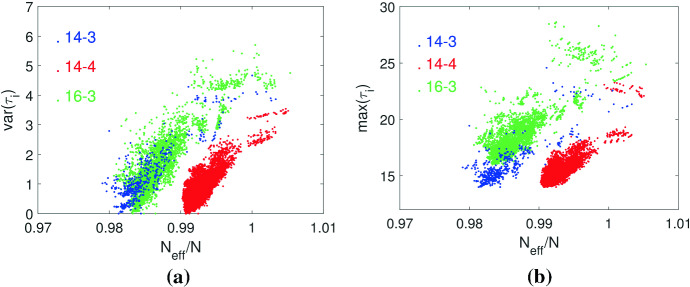
Statistics of remeeting times $\tau _i$ for cubic graphs with $N=\{14,16\}$ and the quartic graphs with $N=14$. Each data point represents a graph. The variance $\text {var}(\tau _i)$ (**a**) and the maximum $\max (\tau _i)$ (**b**) are calculated over the vertices of each graph and given over $N_{eff}/N$, where $N_{eff}$ is the effective population size (1) of the perturbed graph after removing an edge

We next look at how the remeeting times $\tau _i$ are distributed over graphs, see Fig. 2 for results on the cubic graphs with $N=\{14,16\}$ and the quartic graphs with $N=14$. For other *N* the results are similar. The figures show the variance $\text {var}(\tau _i)$ and maximum $\max (\tau _i)$ over $N_{eff}/N$. The average value $\text {ave}(\tau _i)$ is not explicitly shown as from the identity condition $\sum _{i \in {\mathcal {G}}} \pi _{i} ^2\tau _{i}^{}=1$, together with $\pi _i=1/N$ for regular graphs, it follows that $\text {ave}(\tau _i)=N$. We see that amplifier constructors ($N_{eff}/N>1$) have generally large $\text {var}(\tau _i)$ and $\max (\tau _i)$, but these relations are not very strict. In other words, the remeeting times alone allow no clear conclusions about the graph’s ability to become an amplifier constructor. Another result is that the remeeting times of most of these cubic and quartic graphs have non-negligible variance $\text {var}(\tau _i)$. This means a mean-field approximation (Fotouhi et al. 2019), which assumes that the remeeting times have rather equal values, mostly does not apply.

The results so far are for weak selection, that is $$r=1+\delta $$ with $$\delta \rightarrow 0$$. In other words, the graphs that have $$N_{eff}>N$$ are tangential amplifiers. In the following, we check for selected graphs with $$ N_{eff}>N$$ the range $$1<r<r_{max}$$ for which they are transient amplifiers. This is in line with the recent finding that for dB updating there are no universal amplifiers (Tkadlec et al. 2020) and thus there is a finite $$r<r_{max}$$ for which $$\varrho _{{\mathcal {G}}}(r)>\varrho _{{\mathcal {N}}}(r)$$. The study involves calculating the fixation probabilities $$\varrho _{{\mathcal {G}}}(r)$$ by evaluating a Markov state transition matrix, see Sect. 2.1, and comparing them to the fixation probability of the complete graph with *N* vertices, which is given by Eq. (10). Figure 1b shows the result for the quartic graph with order $$N=11$$, which is the smallest graph that is an amplifier constructor, see Table 1. For $$r=1$$ we have $$\varrho _{{\mathcal {G}}}(r)=\varrho _{{\mathcal {N}}}(r)=1/N$$.

**Fig. 3:**
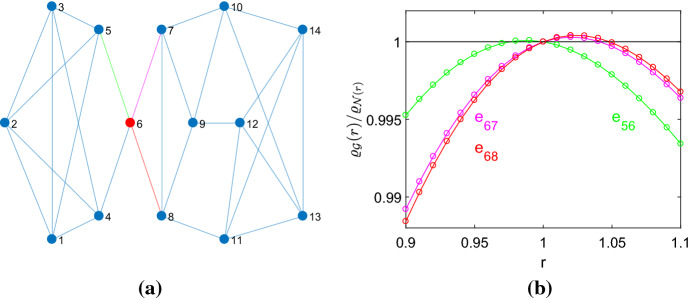
Constructing transient amplifiers from a quartic graph of order $N=14$. **a** The graph with the largest remeeting time $\tau _6=22.0892$ for vertex $v_6$, marked with red. The effective population size $N_{eff}=14.0708$ is obtained by removing the edge $e_{68}$, marked with red, and the removal of the edge $e_{67}$, marked with magenta, yields $N_{eff}=14.0594>N$. Taking away the edges $e_{56}$ or $e_{46}$, however, gives the same value $N_{eff}=13.9705<N$. **b** The quantity $\varrho _{{\mathcal {G}}}(r)/\varrho _{{\mathcal {N}}}(r)$ over *r*. The graphs obtained by removing the edges $e_{68}$ and $e_{67}$ are transient amplifiers (characterized by values $\varrho _{{\mathcal {G}}}(r)/\varrho _{{\mathcal {N}}}(r)>1$ for $1\le r \le r_{max}$), taking away $e_{56}$ or $e_{46}$ yields a behavior, which can be seen as a transient suppressor

For the interval $1<r<r_{max}\approx 1.00075$, the fixation probability of the graph is larger than the fixation probability of the complete graph with $N=11$. As the interval is small with respect to the mutant fitness *r* as well as with respect to the ratio $\varrho _{{\mathcal {G}}}(r)/\varrho _{{\mathcal {N}}}(r)$, it could be suspected that the result might be a numerical artefact. Surely, it could be lengthy and arduous to obtain the results by Monte Carlo simulation as the number of repetitions needed to achieve convergence to the level of required precision would be rather high. However, the results given in Fig. 1b are obtained by exact calculation of the fixation probabilities. An important argument in favor of the validity of the results is that apart from round-off errors the method does not suffer from convergence issues. In order to get an estimation of the round-off error, the calculation of the fixation probabilities is compared to the two cases of regular graphs for which analytical results exist. The comparisons are to the complete graph, which is ($N-1$)-regular, and to the cycle graph, which is 2-regular. For the complete graph the fixation probability is given by Eq. (10), while for the cycle graph, Eq. (11) applies (Kaveh et al. 2015; Hindersin and Traulsen 2015; Allen et al. 2020).

Figure 11 of the Appendix gives the relative error between the calculated fixation probabilities using a Markov state transition matrix and the analytical results for the complete and the cycle graph of order $N=12$ and $N=14$. We see that the round-off error is in the magnitude of $10^{-14}$, while the differences between the fixation probabilities are in the magnitude of $10^{-7}$. Therefore, the results given in Fig. 1b should be regarded as valid.

For the number of vertices *N* increasing, a larger number of regular graphs are amplifier constructors, and also $r_{max}$ and the maximal ratio $\varrho _{{\mathcal {G}}}(r)/\varrho _{{\mathcal {N}}}(r)$ increases. See as another example the quartic graph of order $N=14$ in Fig. 3a, which is the graph with the largest $N_{eff}$ among the ${\mathcal {A}}_4(14)=108$ quartic graph of order $N=14$ with $N_{eff}>14$. A major difference to the example with $N=11$ in Fig. 1a is a lower level of graph symmetry. Thus, it matters which of the edges connecting the vertex with the largest remeeting time ($v_6$) with other vertices is removed. For 2 of the 4 edges we obtain a transient amplifier, but with different $r_{max}$. For the other two edges, we obtain $\varrho _{{\mathcal {G}}}(r)/\varrho _{{\mathcal {N}}}(r)<1$ for $r>1$, but $\varrho _{{\mathcal {G}}}(r)/\varrho _{{\mathcal {N}}}(r)>1$ for some $r_{min}<r<1$. Thus, such a behavior could be seen as a transient suppressor. The Appendix contains further examples of transient amplifiers of cubic graphs with $N=\{18,20,22\}$ and quartic graphs with $N=\{15,16\}$, which each produce the largest $N_{eff}$, see Figs. 13 and 14.

**Fig. 4:**
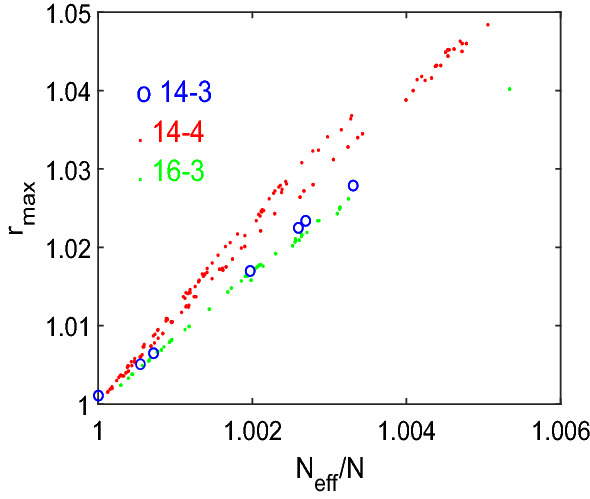
Relationship between $N_{eff}$ and $r_{max}$ for cubic graphs with $N=\{14,16\}$ and quartic graph with $N=14$ with $N_{eff}>N$. The critical mutant fitness $r_{max}$ is calculated from $\varrho _{{\mathcal {G}}}(r)$, which is equidistantly distributed on *r*, by interpolation using a Lagrange polynomial

We now study the relationship between $N_{eff}$ and $r_{max}$, see Fig. 4 showing results for all cubic graphs with $N=\{14,16\}$ and all quartic graph with $N=14$ with $N_{eff}>N$. Again, the results for other *N* are similar. The values of $r_{max}$ are calculated from $\varrho _{{\mathcal {G}}}(r)$ by interpolation using a Lagrange polynomial. There is an almost linear relationship between $N_{eff}/N$ and $r_{max}$. As $N_{eff}$ is the tangential fixation probability at $r=1$, this means that the curve of $\varrho _{{\mathcal {G}}}(r)/\varrho _{{\mathcal {N}}}(r)$ is approximately a parabola, whose intersection with $\varrho _{{\mathcal {G}}}(r)/\varrho _{{\mathcal {N}}}(r)=1$ at $r=r_{max}$ is determined by $N_{eff}$. The results additionally suggest that instead of searching for a large $r_{max}$, we may use $N_{eff}$ as a proxy. As calculating $N_{eff}$ has polynomial time complexity, while calculating $r_{max}$ via $\varrho _{{\mathcal {G}}}(r)$ is exponential, this relationship may save numerical resources. However, it needs to be checked by additional work if the almost linear relationship between $N_{eff}$ and $r_{max}$ is still valid for transient amplifiers that are more strongly perturbed with respect to their regularity as the examples considered here with just a single edge removed.

**Fig. 5:**
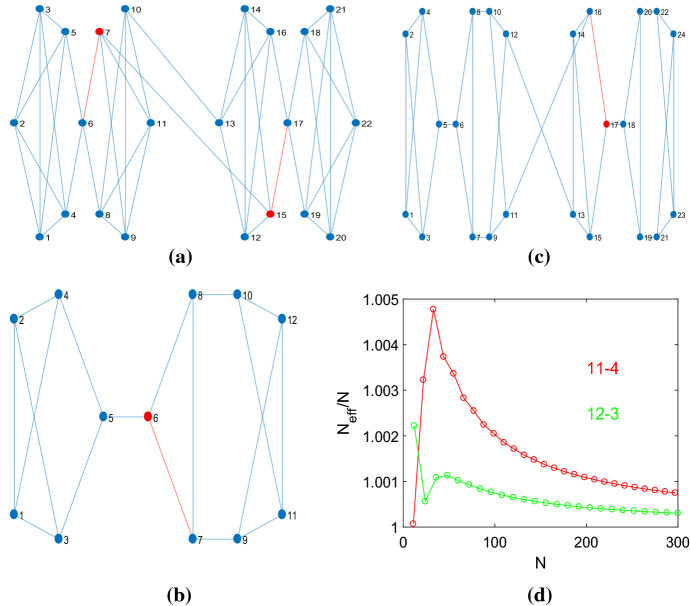
Constructing transient amplifiers by building chains containing a head, a tail and possibly mid-sections. **a** A chain containing a head connected with a tail using the quartic graph of order $N=11$ in Fig. 1a. The resulting chain has largest remeeting times $\tau _7=\tau _{15}=33.9780$, and removing the edge $e_{67}$ or the edge $e_{15\, 17}$ leads to a transient amplifier with $N_{eff}=22.0709>22$. **b** The cubic graph of order $N=12$ that is an amplifier constructor with the largest remeeting time $\tau _6=18.2911$. Removing the edge $e_{67}$ or the edge $e_{68}$ produces $N_{eff}=12.0266>12$, compare to Fig. 2a in Allen et al. (2020). **c** A chain using as building blocks the cubic graph of order $N=12$ shown in (**b**). The largest remeeting time is $\tau _{17}=38.1158$ and removing the edge $e_{16\, 17}$ gives $N_{eff}=24.0134>24$, while removing the edge $e_{15\, 17}$ yields $N_{eff}=23.9161<24$. **d** The quantity $N_{eff}/N$ over *N* for chains with mid-sections. A rising number of mid-section (and thus vertices *N*) gives a series of amplifier constructors. The ratio $N_{eff}/N$ has a peak for a low number of mid-sections and converges to $N_{eff}/N \rightarrow 1$ from above for *N* getting large

The results in Table 1 suggest that amplifier constructors are rare compared to the total number of nonisomorphic regular graphs, but their number might not be limited. There is another argument for assuming that there are arbitrarily many regular graphs that produce transient amplifiers by the perturbation method. Some regular amplifier constructors can be used as building blocks to obtain more amplifier constructors. The simplest example is the quartic graph on $N=11$ vertices shown in Fig. 1a. From this graph another quartic graph on $N=22$ vertices can be obtained by the following procedure. We take two copies of the graph and call them head and tail, respectively. From the head we remove the edge $e_{7 \, 10}$ and from the tail, we remove the edge $e_{24}$. We build a single graph from the head and the tail by retaining the indices of the vertices $(v_1,v_2,\ldots ,v_{11})$ from the head and renaming the vertices of the tail by $(v_{12},v_{13},\ldots ,v_{22}):=(v_1,v_2,\ldots ,v_{11})$. All edges apart from those removed remain unchanged. We finally connect head and tail by additional edges $e_{7\,15}$ and $e_{10\,13}$, see Fig. 5a. From the graph a transient amplifier can be constructed by removing the edge $e_{67}$ (or $e_{15\, 17}$) to obtain $N_{eff}=22.0709>22$.

Another example is the cubic graph in Fig. 5b, which is the only cubic graph of order 12 that is an amplifier constructor, compare also to Fig. 2a in Allen et al. (2020). Here we remove the edge $e_{11\,12}$ from the head and $e_{14}$ from the tail, and connect head and tail by the edges $e_{12 \, 13}$ and $e_{11\,16}$, see Fig. 5c. The use of building blocks can be extended by placing mid-sections between the head and the tail. From these mid-sections we need to remove the edges as for the head and the tail, for instance $e_{7 \, 10}$ and $e_{24}$ for the quartic graph on $N=11$ vertices in Fig. 1a. We then connect the mid-section to the head on the one side and to the tail on the other. Figure 5d shows the ratio $N_{eff}/N$ over *N* for such chains using the building blocks of the quartic graph on $N=11$ vertices, Fig. 1a, and the cubic graph on $N=12$ vertices, Fig. 5b, for $N=\{11,22,\ldots ,297 \}$ and $N=\{12,24,\ldots ,300 \}$. For the number of midsections (and thus the number of vertices) increasing we continue to obtain graphs which are transient amplifiers. However, for *N* getting larger the ratio $N_{eff}/N$ slowly converges to one from above. The perturbation to the regularity of the amplifier constructor loses its impact, and the isothermal theorem (Lieberman et al. 2005) becomes effective.

### Spectral analysis of amplifier constructors

In the previous section it has been argued that regular graphs are a suitable input for a perturbation method to construct transient amplifiers of a dB updating process. In particular, it was shown that a small but significant subset of all pairwise nonisomorphic cubic and quartic regular graphs up to a certain order ($N=22$ for cubic and $N=16$ for quartic) contains amplifier constructors. The results even suggest that the number of amplifier constructors is not limited on the graph order *N* tested by the experimental settings of this paper. As the population structure is expressed by the graph structure this naturally poses this question: Is there something in the structure of these graphs that makes them prone to construct amplifiers? In the following we approach this question by methods of spectral graph theory (Gu et al. 2016; Wilson and Zhu 2008; Wills and Meyer 2020), adding to the applications of spectral analysis of evolutionary graphs (Richter 2017, 2019a, b, 2020; Allen et al. 2019).

**Fig. 6:**
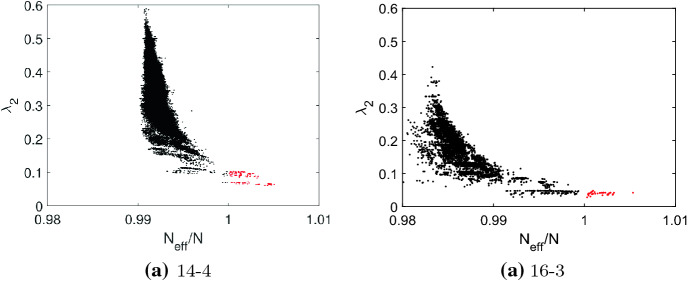
The spectral gap $\lambda _2$ versus the ratio $N_{eff}/N$ as a scatter plot for: **a** the quartic graphs of order $N=14$ and **b** the cubic graphs of order $N=16$. The $N_{eff}$ is the maximal value obtained by the perturbation method removing an edge from the vertex $v_i$ with the largest remeeting time $\tau _i$. Transient amplifiers are marked by red dots. See also Fig. 12 of the Appendix for results of the other *N*

The spectral analysis presented here is based on the *N* eigenvalues $\lambda ({\mathcal {G}})$ of the normalized Laplacian $L_{{\mathcal {G}}}$, which gives us the spectrum $0=\lambda _1\le \lambda _2 \le \ldots \lambda _N\le 2$, see Sect. 2.2. The principal quantity for assessing structural properties of the graph is the spectral gap $\lambda _2$ (Hoffman et al. 2019; Wilson and Zhu 2008; Wills and Meyer 2020). Figure 6 gives the spectral gap $\lambda _2$ over $N_{eff}/N$ for the quartic graphs of order $N=14$ and the cubic graphs with $N=16$ as a scatter plot, for results of the remaining graphs, see the Appendix, Fig. 12. The value of $N_{eff}$ is the maximal value obtained by perturbing the regular graph by removing a single edge from the vertex $v_i$ with the largest remeeting time $\tau _i$. The amplifier constructors producing $N_{eff}/N>1$ are given red dots, while the remaining graphs are indicated by black dots. The main characteristic is that all amplifier constructors have small values of $\lambda _2$. Small values of $\lambda _2$ imply large mixing times, bottlenecks, clusters and low conductance (Banerjee and Jost 2008, 2009; Hoffman et al. 2019; Wills and Meyer 2020). Moreover, a low spectral gap indicates path-like graphs which are rather easy to divide into disjointed subgraphs by removing edges or vertices. In other words, amplifier constructors most likely possess cut and/or hinge vertices (Chang et al. 1997; Ho et al. 1996). There is, however, also a multitude of graphs with low $\lambda _2$ that do not produce transient amplifiers, thus a low spectral gap is necessary but not sufficient for the regular graphs under study to be amplifier constructors. It can be conjectured that this remains true for amplifier constructors with $N>22$. Furthermore, it can be noted that the spectral gap versus $N_{eff}/N$ roughly appears as a half of a parabola with the lowest values of $\lambda _2$ for $N_{eff}/N>1$ and that the majority of values are concentrated for medium values of $\lambda _2$.

**Fig. 7:**
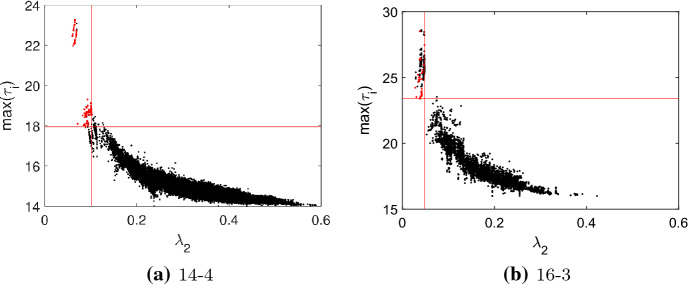
The spectral gap $\lambda _2$ versus the maximum $\max (\tau _i)$ as a scatter plot for: **a** the quartic graphs of order $N=14$ and **b** the cubic graphs of order $N=16$. Transient amplifiers are marked by red dots. The red lines indicate the smallest $\max (\tau _i)$ and the largest $\lambda _2$ for which a quartic graph with $N=14$ and cubic graph with $N=16$, respectively, are amplifier constructors. These values are used for a linear classifier to identify amplifier constructors

**Fig. 8:**
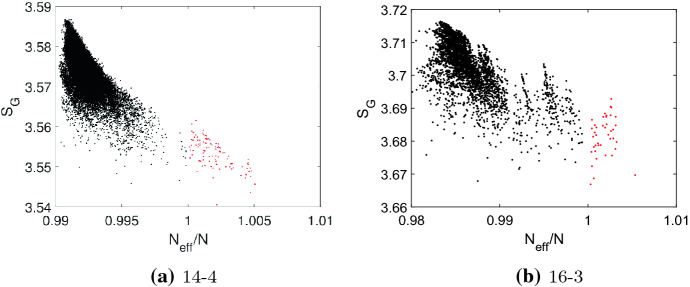
The von Neumann entropy $S_{{\mathcal {G}}}$ versus the ratio $N_{eff}/N$ as a scatter plot for: **a** the quartic graphs of order $N=14$ and **b** the cubic graphs of order $N=16$. The $N_{eff}$ is the maximal value obtained by the perturbation method removing an edge from the vertex $v_i$ with the largest remeeting time $\tau _i$. Transient amplifiers are marked by red dots

**Fig. 9:**
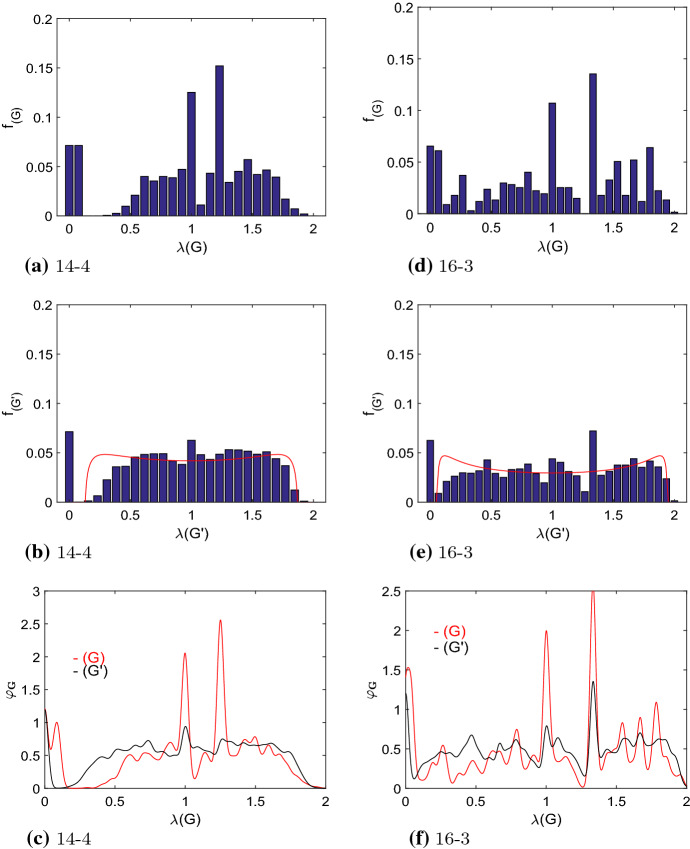
The discrete eigenvalue distributions $f_{(G)}$ of the amplifier constructors ($N_{eff}/N>1$) and $f_{(G')}$ for the remaining graphs with ($N_{eff}/N<1$) and the smoothed spectral densities $\varphi _{\mathcal {(G)}}$ and $\varphi _{\mathcal {(G')}}$ according to Eq. (12) for all quartic regular graphs with $N=14$, **a**–**c**, and all cubic regular graphs with $N=16$, **d**–**f**. The red lines in (**b**) and (**e**) depict the shape of the Kesten–McKay distribution (16), which is rescaled in order to cover the same area as the bars of the histogram. The spectral density of the amplifier constructors is shown as red line in (**c**) and (**f**), while the black line is for the remaining graphs. See also Fig. 15 of the Appendix for results of the smoothed spectral densities $\varphi _{\mathcal {(G)}}$ and $\varphi _{\mathcal {(G')}}$ for the other *N*

Recently, in the context of evolutionary games on isothermal graphs a bound on the remeeting times $\tau _i$ has been proven (Allen et al. 2019). Although the spectral gap of the adjacency matrix *A* was studied rather than the spectral gap of the normalized Laplacian $L_{{\mathcal {G}}}$, which is considered here, the findings equivalently apply as for regular graphs $L_{{\mathcal {G}}}=I-1/k \cdot A$, and thus the spectral gaps directly relate to each other. The reverse is not necessarily true as all regular graphs are isothermal but not the other way around. For regular graphs, the bound on remeeting times applies equivalently for the spectral gaps $\lambda _2$ of *A* and $L_{{\mathcal {G}}}$, and we have (Allen et al. 2019)

15 $$\begin{aligned} \tau _i \le \frac{N-1}{\lambda _2}+\frac{2N-1}{N}. \end{aligned}$$

From this bound it follows that graphs with large $\max (\tau _i)$ must necessarily have small $\lambda _2$. The converse is not necessarily true. Figure 7 examines this relationship and gives $\max (\tau _i)$ versus $\lambda _2$ as a scatter plot for all quartic and cubic regular graphs with $N=\{14,16\}$. In addition, it is indicated by color whether or not the graphs are amplifier constructors. The results show that many amplifier constructors are effectively characterized by the combination of large values of $\max (\tau _i)$ and small value of $\lambda _2$. However, there are also exceptions from this rule as for some graphs the connections between $N_{eff}/N$ and the spectral gap are not mediated through $\max (\tau _i)$.

An alternative characterization of Laplacian spectra is based on an entropic graph measure. The results obtained for the von Neumann entropy $S_{{\mathcal {G}}}$ as defined by Eq. (14) versus $N_{eff}/N$ are given in Fig. 8. The results are for the quartic graphs of order $N=14$ and the cubic graphs with $N=16$ and shown as a scatter plot, also compare to the results for the spectral gap $\lambda _2$, Fig. 6. The results for the remaining graphs are very similar. Larger values of the von Neumann entropy $S_{{\mathcal {G}}}$ indicate a growing number of connected components, long paths and nontrivial symmetries, while smaller values are connected with short paths and large cliques (Passerini and Severini 2009; Du et al. 2010; Han et al. 2012; Feng et al. 2019; Minello et al. 2019). The results in Fig. 8 show that amplifier constructors generally have smaller values of $S_{{\mathcal {G}}}$ as compared to graphs which do not produce transient amplifiers, but similarly to the spectral gap $\lambda _2$ a low Laplacian entropy $S_{{\mathcal {G}}}$ is only necessary but not sufficient.

**Fig. 10:**
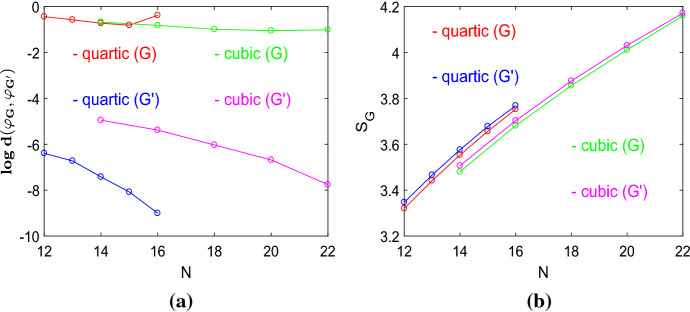
Comparing regular graphs that construct amplifiers (cubic graphs as green line, quartic graphs as red line) with graphs that do not (cubic graphs as magenta line, quartic graphs as blue line). **a** The spectral distance measure $d({\mathcal {G}},{\mathcal {G}}')$ defined by Eq. (13) on logarithmic scale. **b** The averaged von Neumann entropy $S_{\mathcal {G}}$ defined by Eq. (14)

As it seems unlikely that a single spectral measure (or any other scalar graph measure such as average path length or clustering coefficient) can uniquely identify an amplifier constructor, we next look at the whole spectrum. Figure 9 shows the spectral distributions for the quartic graphs of order $N=14$ and the cubic graphs with $N=16$. The upper four panels give the discrete eigenvalue distributions $f_{(G)}$ of the amplifier constructors ($N_{eff}/N>1$) and $f_{(G')}$ for the remaining graphs with $N_{eff}/N<1$, while the lower panels show the smoothed spectral densities $\varphi _{\mathcal {(G)}}$ and $\varphi _{\mathcal {(G')}}$ according to Eq. (12). The smoothed spectral density $\varphi _{\mathcal {(G)}}$ for the remaining graphs is given in the Appendix, Fig. 15. In all these figures the spectral density of the amplifier constructors $\varphi _{\mathcal {(G)}}$ is shown as red line, while the black line is for the remaining graphs ($\varphi _{\mathcal {(G')}}$).

**Fig. 13:**
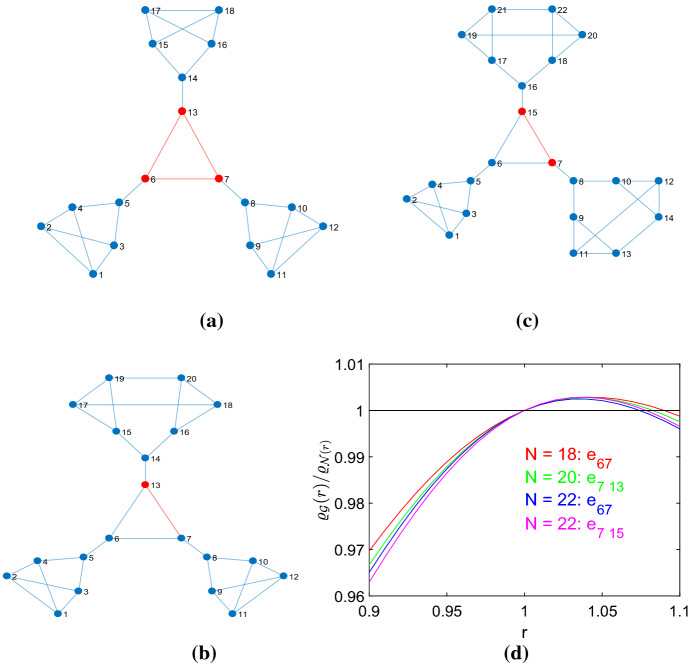
Cubic amplifier constructor graphs producing the largest $N_{eff}$ among all ${\mathcal {A}}_3(N)$ amplifier constructors according to Table 1. **a** $N=18$: Largest remeeting times for all three vertices in the triangle center of the graph, $\tau _6=\tau _7=\tau _{13}=31.9937$. By removing either the edge $e_{67}$ or $e_{6\,13}$ or $e_{7\,13}$ we get $N_{eff}=18.2721$. **b** $N=20$: The largest remeeting time is $\tau _{13}=36.9143$ and removing the edge $e_{6\,13}$ or $e_{7\,13}$ yields $N_{eff}=20.2884$. **c** $N=22$: The largest remeeting times are $\tau _7=\tau _{15}=40.8256$ and removing the edge $e_{7\,15}$ yields $N_{eff}=22.3149$, while removing the edge $e_{67}$ or $e_{6\,15}$ gives $N_{eff}=22.2873$. **d** The quantity $\varrho _{{\mathcal {G}}}(r)/\varrho _{{\mathcal {N}}}(r)$ over *r*. Values of $\varrho _{{\mathcal {G}}}(r)/\varrho _{{\mathcal {N}}}(r)>1$ for $r>1$ indicate a transient amplifier

**Fig. 14:**
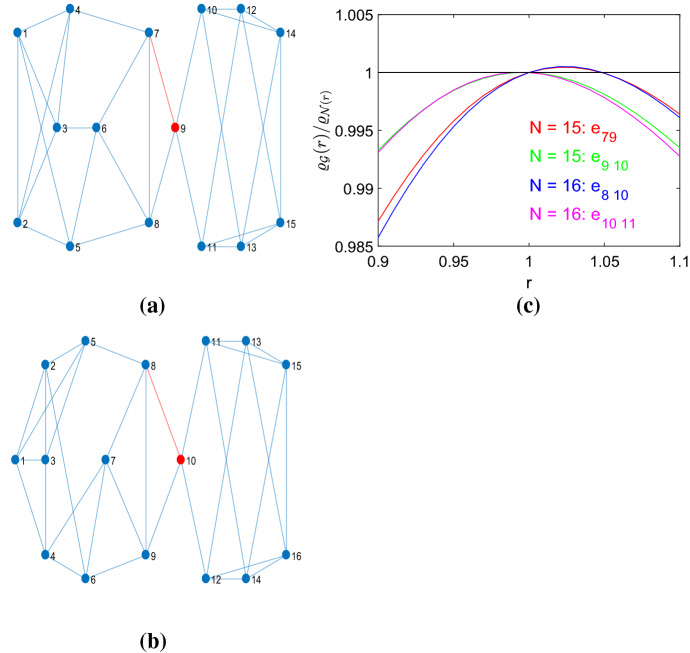
Quartic amplifier constructor graphs producing the largest $N_{eff}$ among all ${\mathcal {A}}_4(N)$ amplifier constructors according to Table 1. **a** $N=15$: Largest remeeting times for vertex $v_9$ with $\tau _9=24.3560$. By removing either the edge $e_{79}$ or $e_{89}$ we get $N_{eff}=15.0787$ or $N_{eff}=15.0675$ and a transient amplifier, while removing $e_{9 \, 10}$ or $e_{9\,11}$ gives $N_{eff}=14.9991$ and a fixation probability $\varrho _{{\mathcal {G}}}(r)$ that is always smaller than $\varrho _{{\mathcal {N}}}(r)$ of the complete graph except for $r=1$, where it equals. **b** $N=16$: The largest remeeting time is $\tau _{10}=25.8981$ and removing the edge $e_{8\,10}$ yields $N_{eff}=16.0883$, while removing $e_{10\,11}$ or $e_{10\,12}$ only yields $N_{eff}=15.9833$. **c** The quantity $\varrho _{{\mathcal {G}}}(r)/\varrho _{{\mathcal {N}}}(r)$ over *r*. Values of $\varrho _{{\mathcal {G}}}(r)/\varrho _{{\mathcal {N}}}(r)>1$ for $r>1$ indicate a transient amplifier

**Fig. 15:**
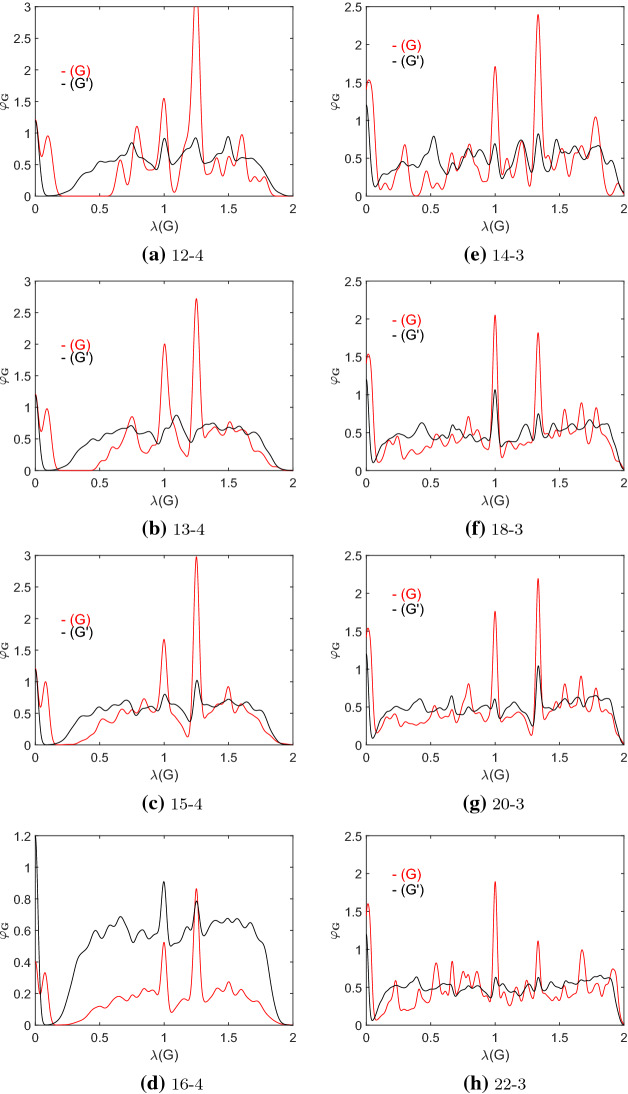
The smoothed spectral density $\varphi _G$ for amplifier constructors (red line) and the remaining regular graphs (black line) for: **a**–**d** the quartic graphs of order $N=\{12,13,15,16\}$ and **e**–**h** the cubic graphs of order $N=\{14,18,20,22\}$. Supplement to Fig. 9

We can now compare the results for amplifier constructors with other regular graphs. Most noticeable the spectra of amplifier constructors (upper panels of Fig. 9) differ substantially from the remaining graphs (middle panels of Fig. 9) and also from random regular graphs in general (Farkas et al. 2001; Oren et al. 2009; Bauerschmidt et al. 2019). For the limit case of $N \rightarrow \infty$, the spectral density of random regular graphs can be described by the Kesten–McKay distribution (McKay 1981; Oren et al. 2009; Bauerschmidt et al. 2019), which for the normalized Laplacian and regular graphs is

16 $$\begin{aligned} \varphi _{KM}(x)= \frac{\sqrt{4(k-1)-k^2(1-x)^2}}{2 \pi k (2-x)x} \end{aligned}$$

with $1-2\sqrt{k-1}/k \le x \le 1+2\sqrt{k-1}/k$. The distribution $f_{(G')}$ and the spectral density $\varphi _{\mathcal {(G')}}$ obtained for the regular graphs that are not amplifier constructors have at least some similarity to the Kesten–McKay distribution, see the shape of the Kesten–McKay distribution given in the middle panels of Fig. 9. The distribution $f_{(G)}$ and the spectral density $\varphi _{\mathcal {(G)}}$ obtained for amplifier constructors do not. According to the Kesten–McKay law (16) the eigenvalue density is evenly and symmetrically distributed on the interval [0, 2]. For low values of *k* we find two maxima close to the upper and lower limit of the distribution. For amplifier constructors the spectra do not conform to such characteristics. We find two characteristic peaks, one at $\lambda _i=1$ and another at $1<\lambda _i<1.5$. This can be found for all cubic and quartic regular graphs tested, see also Fig. 15. These peaks indicate a substantial multiplicity of the eigenvalues. Multiplicity of the eigenvalues of the normalized Laplacian is connected to motif doubling and motif attachment, which can be seen as the process of building a graph from joining or repeating identical substructures (Banerjee and Jost 2008, 2009; Mehatari and Banerjee 2015). In other words, amplifier constructors are graphs which with a high probability contain identical (or at least almost identical) subgraphs and thus exhibit a substantial degree of graph symmetry. This conclusion is certainly exemplified by the results for the graphs producing highest $N_{eff}$ given in Figs. 13 and 14.

As the spectral density of amplifier constructors differs from other regular graphs, we next look at the spectral distance measure $d({\mathcal {G}},{\mathcal {G}}')$, see Eq. (13), to capture and quantify the differences in the graph structure, see Fig. 10a. The distance measure is given for all cubic graphs with $N=\{14,16,18,20,22\}$ and all quartic graphs with $N=\{12,13,14,15,16\}$. The distances are calculated by comparing the amplifier constructors (*G*) of a given order *N* and degree *k* to all graph with the same order and degree. The same is done for the graphs $(G')$ which are not amplifier constructors. We see that amplifier constructors have much higher spectral distances, which remains largely constant for *N* getting larger. By contrast, graphs that are not amplifier constructors have a low spectral distance, are very similar to the set of all regular graphs with given *N* and *k*, and the distance gets small for *N* increasing. This can be explained by the increasing number of pairwise nonisomorphic graphs for *N* getting larger [see Table 2 for the values of ${\mathcal {L}}_k(N)$]. It means that the fraction of graphs that are structurally similar increases as well. It is also interesting to note that the spectral distances for quartic graphs are lower than for cubic graphs. A possible interpretation is that cubic regular graphs show a larger structural variety than quartic regular graphs. To conclude amplifier constructors can be clearly distinguished from regular graphs that do not yield transient amplifiers by their spectral density profile. To use the language of data mining and interpreting the spectral density as a data set, there is a characteristic and discoverable structure in the spectra of amplifier constructors which enables to identify them.

Finally, we compare the von Neumann entropy $S_{{\mathcal {G}}}$ as defined by Eq. (14) averaged over all cubic graphs with $N=\{14,16,18,20,22\}$ and all quartic graphs with $N=\{12,13,14,15,16\}$, but differentiating amplifier constructors (*G*) and graphs $(G')$ which are not amplifier constructors, see Fig. 10b. Similar to the spectral distance measure $d({\mathcal {G}},{\mathcal {G}}')$, we can distinguish both groups of graphs. Amplifier constructors have generally smaller values of the averaged von Neumann entropy $S_{{\mathcal {G}}}$, which indicates that they tend to have shorter paths and larger cliques.

## Discussion

### Constructing transient amplifiers

Transient amplifiers of selection are structured networks that increase the fixation probability of beneficial mutations as compared to a well-mixed population. Thus, transient amplifiers provide a mechanism for shifting the balance between natural selection and genetic drift, and therefore have considerable significance in evolutionary dynamics (Allen et al. 2020; Hindersin and Traulsen 2015; Pavlogiannis et al. 2018; Tkadlec et al. 2020). Until recently, it was assumed that transient amplifiers are rare or nonexistent for death–birth (dB) updating. In a recent work (Allen et al. 2020) first examples of transient amplifiers for dB updating were presented together with a procedure executable with polynomial time complexity to decide whether or not a given graph is an amplifier of weak selection. This procedure includes finding the vertex with the largest remeeting time and perturbing the graph by removing an edge from this vertex. There is a certain likelihood that the resulting perturbed graph is a transient amplifier. This paper extends the approach by using the perturbation method on regular graphs up to a certain order. We check all pairwise nonisomorphic regular graphs of order $N \le 14$ and in addition 3-regular with $N\le 22$ and 4-regular graphs with $N\le 16$. The results show that a small but significant subset of cubic and quartic regular graphs produce perturbed graphs that are transient amplifiers. The regular graphs possessing this property are called amplifier constructors.

Thus, a first major finding of this study is that most likely transient amplifiers for dB updating are not as rare as previously assumed. Although the percentage of amplifier constructors is low (for instance $0.19\%$ for cubic graphs of order $N=22$ or $0.04\%$ for cubic graphs of order $N=16$), the exponential growth with order *N* of the number of pairwise nonisomorphic graphs ensures their number is not negligible. The given percentages mean ${\mathcal {A}}_3(22)=13.889$ and ${\mathcal {A}}_4(16)=3.129$ amplifier constructors for the cubic and quartic graphs of order $N=22$ and $N=16$, respectably. As for the considered order *N* the exact number of all connected pairwise nonisomorphic cubic and quartic graphs is known, and all instances have been tested, the study encompasses the whole structural range of these graphs. In other words, if different graph structures constitute the space of different population structures, all possible structural variants expressible by regular graphs have been covered by this study.

The results also show that certain evolutionary graphs may have interesting and desirable properties (for instance being transient amplifiers) which are rare with respect to the total number of structurally different graphs from a certain class of graphs. This may be relevant for conducting numerical studies which use a rather small set of algorithmically generated graphs, for instance random graphs based on the models of Erdös–Rényi, Barabási–Albert or Watts–Strogatz. There is a significant chance that a study based on such a set of graphs may fail to identify such a rare property.

The perturbation methods discussed and analyzed here only removes a single edge. Thus, the difference between a transient amplifier produced by the perturbation and a regular graph is small. This may also explain why the effective population size $N_{eff}$ of the transient amplifiers remains close to *N* and as a consequence also the maximal fitness $r_{max}$ for which amplification occurs is relatively close to $r=1$. Possibly, a modification of the perturbation method that systematically removes a larger number of edges would also yield larger $r_{max}$. Moreover, the transient amplifiers considered here are based on graphs that are not weighted. As unweighted graphs restrict the effect of substantial amplifications (Pavlogiannis et al. 2018; Tkadlec et al. 2019), combining the perturbation method with designing weights may also lead to larger values of $r_{max}$. Both, constructing transient amplifiers by systematically removing several edges from a regular graph and designing weights may be topics for future work.

The analysis given in this paper focuses on cubic and quartic regular graphs. This focus is not due to computational reasons in the sense that graphs with another degree cannot be handled numerically. The main reason is the fact that removing an edge from regular graphs with a larger degree does not produce transient amplifiers for an order up to 14 (with the exception of a tiny percentage of quintic graphs with order 14). Also for ($N-3$)-regular graphs of order $N=\{15,16,18,20\}$ no amplifier constructors have been found. This is interesting and becomes plausible by looking at the perturbation method and particularly at the approximation given by Eq. (9). Suppose a vertex *m* has the largest remeeting time $\tau _m$ and we perturb the graph by removing the edge to the vertex *n*, thus lowering the degree of these two vertices. Consequently, $\varDelta \pi _m$ and $\varDelta \pi _n$ become negative, while every $\varDelta \pi _i$ of the remaining vertices becomes positive. For obtaining a positive $\varDelta N_{eff}$, and thus a transient amplifier, we need the negative sum of $(\varDelta \pi _m\tau _m+ \varDelta \pi _n\tau _n)$ to outweigh the sum of $\varDelta \pi _i \tau _i$ for the remaining vertices. For regular graphs with degree 3 and 4 this occurs, rarely but not unprecedentedly, as shown in the paper. Computationally, it is possible because some of these regular graphs have a rather large variance $\text {var}(\tau _i)$ and the magnitude of the negative $\varDelta \pi _i$ is rather large as well. By contrast, for graphs with a higher degree the variance $\text {var}(\tau _i)$ is smaller and removing an edge leads to a smaller magnitude of negative $\varDelta \pi _i$. These two properties prevent the sum of $\varDelta \pi _i \tau _i$ from becoming negative, and thus regular graphs with a larger degree produce no transient amplifier, at least not for the *N* considered, but quite likely it is a general property. This also becomes plausible by interpreting these graphs as interaction networks. Transient amplifiers are networks which have a higher fixation probability than complete graphs for beneficial mutations within the range $1<r<r_{max}$. This property is connected with the graph possessing clusters that are connected by a small number of vertices. A perturbation of a regular graph creates a transient amplifier if removing the edge weakens the connection between these clusters and thus reinforces a bottleneck. However, above a certain graph degree and thus a certain number of neighbors for each vertex, it becomes impossible to create a bottleneck by removing a single edge, because there are enough edges left to counter the effect. Thus, regular graphs above a certain degree do not produce transient amplifiers by such an edge-removing perturbation.

### Spectral analysis

A spectral analysis revealed that the amplifier constructors share certain structural properties. The low spectral gap indicates that these graphs are path-like and can be easily divided into subgraphs by removing a low number of edges and/or vertices. From a graph-theoretical point of view, amplifier constructors most likely have cut and/or hinge vertices (Chang et al. 1997; Ho et al. 1996). The instances of the transient amplifiers producing the largest $N_{eff}$, see Figs. 13 and 14 in the Appendix, are illustrative examples for this property. For instance, the graphs producing the highest $N_{eff}$ for the cubic graphs, see Fig. 13, have a triangular hub as a center, while each of the three vertices forming the triangular hub is a cut vertex. This is in line with results showing low values of the von Neumann graph entropy for amplifier constructors, which also points at short paths and large cliques.

The analysis of the spectral density of the amplifier constructors has further shown a multiplicity of the eigenvalues of the normalized Laplacian. This spectral property is connected to motif doubling and motif attachment, which can be seen as the process of building a graph from joining or repeating identical substructures (Banerjee and Jost 2008, 2009; Mehatari and Banerjee 2015). This allows the conclusion that with a high probability amplifier constructors contain identical (or at least almost identical) subgraphs and thus exhibit a substantial degree of graph symmetry. Also this can be clearly seen in the graphs producing highest $N_{eff}$, see Figs. 13 and 14. It might be interesting to note that similar results have been reported for other biological networks, for instance metabolistic networks, transcription networks, food-webs and phylogenetic trees (Banerjee and Jost 2007; Lewitus and Morlon 2016; Milo et al. 2002). This suggests the speculation that motif doubling and motif attachment is a general property of mechanisms in biological networks, which also manifests itself in the interplay between the graph structure and the evolutionary dynamics of mutants invading a network. In this respect, the graph structures identified by Allen et al. (2020) and denoted as fans, separated hubs and stars of islands are also fitting these structural patterns. Some of these graphs can be turned into transient amplifiers for dB updating by designing weights for certain edges. Each of these three structures consists of a hub with either one vertex (the fan), or several non-adjacent vertices (the separated hub), or several adjacent vertices (the star of islands). The hub is surrounded by blades which are cliques of arbitrary size, for instance triangles of vertices. The number of these blades may vary. Clearly, such structures can be build by motif doubling and motif attachment (the motif being the triangular blade) and quite likely a spectral analysis would yield similar results as given here. Similar considerations also apply to other graph structures identified as (transient) amplifiers such as (super-) stars and comets (Jamieson-Lane and Hauert 2015; Pavlogiannis et al. 2017).

It has been shown by Allen et al. (2019) that the remeeting times are bounded depending on the spectral gap $\lambda _2$ by Eq. (15). From this bound it follows that graphs with a large maximal remeeting time $\max (\tau _i)$ must necessarily have a small spectral gap $\lambda _2$, which also applies to amplifier constructors, see Fig. 7. These results suggest using the data set of $\max (\tau _i)$ and $\lambda _2$ for a classifier-based identification of amplifier constructors. Take as a very simple example a linear classifier which identifies a graphs as an amplifier constructor if $\max (\tau _i)$ is above and $\lambda _2$ is below certain thresholds. Define the thresholds as the smallest $\max (\tau _i)$ and the largest $\lambda _2$ for which a graphs with given order and degree is an amplifier constructor. Applying such a linear classifier we obtain, for instance, for the cubic graphs of order $N=16$ in addition to the 42 true positives (graphs correctly identified as amplifier constructors) 139 false positives (graphs that have values above and below the respective thresholds, but are not producing transient amplifiers). Thus, we have a precision (or positive prediction value) of $\text {PRE}=42/(139+42)=23.2044 \%$. Table 3 in the Appendix gives the performance (in $\%$) of such a linear classifier by precision PRE and accuracy ACC for all cubic and quartic graphs tested. The precision PRE is defined as the fraction of all correctly classified amplifier constructors (true positives over all graphs identified as amplifier constructor), while the accuracy is the fraction of all correctly classified graphs (true positives plus true negatives over all graphs). The performance is generally good, particularly if accuracy is considered, which remains high for all *N* tested. On the other hand, precision deteriorates for *N* getting larger, which is most visible for cubic graphs, while for quartic graphs the performance is more stable. This most likely is again the effect of differences in the structural variety between cubic and quartic graphs, and differences in the percentage of amplifier constructors. Overall, the performance of the linear classifier shows that the relationship between the maximal remeeting time $\max (\tau _i)$ and the spectral gap $\lambda _2$ is strong and particularly effective for identifying graphs which have not the property that they can be perturbed into transient amplifiers. In other words, such a classification may be useful to pre-select candidates for amplifier constructors. Quite possibly, the classification performance could be improved by more sophisticated schemes, such as support vector machines, kernel estimation, or machine learning based on neural networks. Such schemes could also be augmented by employing additional graphs measures such as graph entropy or others.

The graphs identified in this paper as amplifier constructors have, generally speaking, no expander properties. This is in contrast to the findings of a recent work which deals with evolutionary games on isothermal graphs, analyses relationships to the spectral gap of the graphs and discusses an intriguing link to expanders (Allen et al. 2019). For these evolutionary games it was shown that the prevalence of cooperative behavior is connected to the effective degree for random isothermal graphs. Moreover, it was demonstrated that there are bounds on the effective degree in terms of the spectral gap for expander graphs. In other words, expander properties might be particularly relevant for understanding cooperative behaviour on graphs. The results given in this paper do not point at expanders, which suggests some speculations. Isothermal graphs neither amplify nor suppress the effect of selection, as do regular graphs. As demonstrated here, perturbing regular graphs by having a single edge removed from a regular graph does yield transient amplifiers. It might be that those “weakly” disturbed and thus “almost regular” graphs require different structural properties as random isothermal graphs which usually have a lesser degree of regularity. Also these relationships should be addressed by future work.

## Conclusions

Transient amplifiers are prominent examples of evolutionary graphs that are capable of increasing the effect of selection and suppressing random drift. Thus, identifying and analyzing these structures is of considerable interest for understanding evolutionary dynamics of biological processes. In the paper it is shown that transient amplifiers for dB updating can be constructed from certain *k*-regular graphs. Particularly, for cubic and quartic graphs we find a sufficiently large set of transient amplifiers for a rather small graph order. Moreover, these amplifiers share certain structural properties. They are rather path-like graphs with low conductance which are relatively easy to divide into subgraphs by removing edges and/or vertices. Frequently, the subgraphs are identical (or at least very similar) and can be viewed as building blocks which are connected by cut and/or hinge vertices. This suggest the question of why and how these structural properties promote the spread of beneficial mutations. A possible explanation comes from viewing these structural properties from the point of evolutionary dynamics on the graph. The dynamics of random walks on graphs with these structural properties implies large mixing times, bottlenecks and the emergence of clusters. On the other hand, it is known that clusters of mutants (or cooperators in the case of evolutionary games) promote survival and facilitate spatial invasion, as shown for lattice grids (Hauert 2001; Hauert and Doebeli 2004; Langer et al. 2008; Page et al. 2000), circle graphs (Xiao and Wu 2019) and selected regular graphs (Richter 2019b). Thus, it appears plausible that for this reason the structural properties identified for amplifier constructors also promote the spread of beneficial mutations.

These explanations also agree with the observation that regular graphs with a degree above a certain value do not possess the property that an edge-removing perturbation produces a transient amplifier. Thus, the results of the paper show that evolutionary graphs to be perturbed into selection amplifiers for birth-death updating require a rather low number of neighbors. This is a reminiscence of an important result in evolutionary game theory (Lieberman et al. 2005), which shows that cooperation thrives when each individual in a network has just a few neighbors. Together with the spectral analysis showing further graph-theoretical properties, the results advance our knowledge about potential network characteristics of selection amplifiers. Given the relevance of selection amplifiers for understanding evolutionary dynamics, such a knowledge may foster analyzing and detecting such networks. Moreover, searching for structural graph properties could also guide the design process of transient amplifiers. This may mean either looking for graphs with prescribed spectral characteristics, or directing algorithms generating random graphs towards the relevant structural patterns. Additional work is needed to further clarify these relationships.

The results of this paper may also be of interest beyond computational biology as amplifiers and suppressors of natural selection have also significant implications on our knowledge about the interplay between the population structure and evolutionary dynamics of real biological processes. How spatial population structure influences evolutionary dynamics has, for instance, been shown for cancer initiation and progression (Hindersin et al. 2016b; Komarova et al. 2003; Komarova 2006; Nowak et al. 2003; Vermeulen et al. 2013), ageing of tissues (Cannataro et al. 2016, 2017), the spread of infections (Ottino-Löffler et al. 2017a, b) and the microbial evolution of antibiotic resistance (Krieger et al. 2020). Furthermore, a direct biotechnological application of the construction of amplifiers has been suggested with in vitro evolution, where amplifiers could help to discover optimized protein or nucleotide sequences with selected medical or biotechnical functions (Pavlogiannis et al. 2018; Möller et al. 2019).

## Figures and Tables

**Fig. 11:**
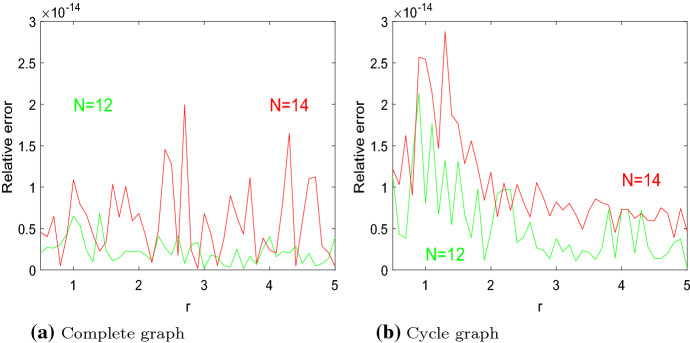
The relative error between the numerical computation of the fixation probability using a Markov state transition matrix and the analytical result over mutant fitness *r* for $N=12$ and $N=14$. **a** For the complete graph with the analytical result according to Eq. (10). **b** For the cycle graph with analytical result according to Eq. (11)

**Fig. 12:**
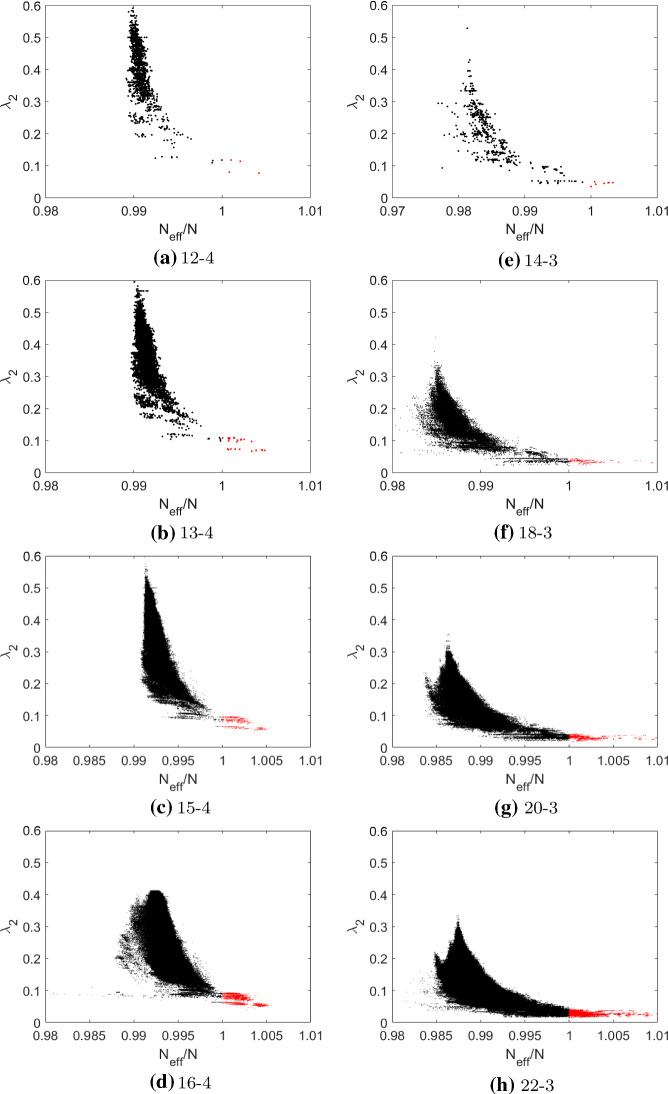
The spectral gap $\lambda _2$ versus the ratio $N_{eff}/N$ as a scatter plot for: **a**–**d** the quartic graphs of order $N=\{12,13,15,16\}$ and **e**–**h** the cubic graphs of order $N=\{14,18,20,22\}$. Supplement to Fig. 6

## Appendix

The results of this paper are calculated and visualized with MATLAB. The adjacency matrices of the set of all amplifier constructors as well as code to produce the results are available at

https://github.com/HendrikRichterLeipzig/TransientAmplifiersRegularGraphs.

Additional graphs and tables with the numbers ${\mathcal {L}}_k(N)$ of simple connected pairwise nonisomorphic *k*-regular graphs on *N* vertices and the performance of a linear classifier to identify amplifier constructors are given in the following Appendix (Figs. 11, 12, 13, 14 and 15; Tables 2 and 3).

**Table 2:** The numbers ${\mathcal {L}}_k(N)$ of simple connected pairwise nonisomorphic *k*-regular graphs on *N* vertices with degree $k\le 6$ used in this study, see e.g. Meringer (1999) and WolframMathWorld (2021)

*N*	k			
	3	4	5	6
11	0	265	0	266
12	85	1.544	7.848	7.849
13	0	10.778	0	367.860
14	509	88.168	3.459.383	21.609.300
15	0	805.491		
16	4.060	8.037.418		
18	41.301			
20	510.489			
22	7.319.447			

The numbers ${\mathcal {L}}_k(N)$ corresponds to the number of regular interaction networks with *N* individuals and *k* interacting neighbors for $11 \le N \le 22$

**Table 3:** Performance of a linear classifier identifying amplifier constructors from maximal remeeting time $\max (\tau _i)$ and spectral gap $\lambda _2$ for cubic and quartic regular graphs

*N*	k			
	3		4	
	PRE	ACC	PRE	ACC
12	100	96.4706	100	99.8705
13			95.8333	99.9351
14	41.1765	95.6778	90.8333	99.9297
15			85.5403	99.3778
16	23.2044	96.4286	70.5524	99.8950
18	20.6547	96.8887		
20	14.5562	95.2442		
22	6.1967	97.7082		

The results are the precision PRE and the accuracy ACC in $\%$. The precision PRE is defined as the fraction of all correctly classified amplifier constructors (true positives over all graphs identified as amplifier constructor), while the accuracy is the fraction of all correctly classified graphs (true positives plus true negatives over all graphs)
